# Size confinement of Si nanocrystals in multinanolayer structures

**DOI:** 10.1038/srep17289

**Published:** 2015-11-25

**Authors:** Rens Limpens, Arnon Lesage, Minoru Fujii, Tom Gregorkiewicz

**Affiliations:** 1 Van der Waals-Zeeman Institute, University of Amsterdam, 1098 XH Amsterdam, The Netherlands; 2 Department of Electrical and Electronic Engineering, Graduate School of Engineering, Kobe University, Rokkodai, Nada, Kobe 657-8501, Japan

## Abstract

Si nanocrystals (NCs) are often prepared by thermal annealing of multiple stacks of alternating sub-stoichiometric SiO_*x*_ and SiO_2_ nanolayers. It is frequently claimed that in these structures, the NC diameter can be predefined by the thickness of the SiO_*x*_ layer, while the NC concentration is independently controlled by the stoichiometry parameter *x*. However, several detailed structural investigations report that the NC size confinement to within the thickness of the SiO_*x*_ layer is not strictly obeyed. In this study we address these contradicting findings: based on cross-correlation between structural and optical characterization of NCs grown in a series of purposefully prepared samples of different stoichiometry and layer thickness, we develop a comprehensive understanding of NC formation by Si precipitation in multinanolayer structures. We argue that the narrow NC size distribution generally observed in these materials appears due to reduction of the Si diffusion range, imposed by the SiO_2_ spacer layer. Therefore, both the SiO_*x*_ layer thickness and composition as well as the actual thickness of the SiO_2_ spacer play an essential role in the NC formation.

Since the discovery of quantum confinement enhanced emission from Si^1^,^2^,^3^, Si nanocrystals (NCs) have received a great deal of attention - see^4^ for a recent review. They can be produced by a variety of experimental techniques, both top-down and bottom-up. In particular, Si NCs of high optical quality, *i.e.* with bright and tunable emission, can be formed by thermal annealing of sub-stoichiometric SiO_*x*_ layers, with 1 ≤ *x* ≤ 2. Layer preparation methods include sputter deposition^5^,^6^, ion beam implantation^7^, laser ablation, plasma-enhanced chemical vapor deposition^6^,^8^, commercial powder^9^ and hydrogen silsesquioxane annealing^10^. Unfortunately, in thermal annealing of sub-stoichiometric SiO_*x*_ layers, the NC size and density are mutually entangled. The disadvantage follows inherently from the fact that the method is based on self-organization of the supersaturated solution of Si within SiO_2_, and the NC size and density are both simultaneously influenced by the Si excess (given by the sub-stoichiometry parameter *x*) and the annealing process (temperature and duration). One way to alleviate this problem is by a preparation of multilayer (ML) stacks of alternating nanolayers of sub-stoichiometric SiO_*x*_ and SiO_2_ 6,11,12,13,14,15,16,17,18,19,20. The underlying idea is that, upon annealing, NC formation is restricted to the SiO_*x*_ layers, thus limiting the NC growth and predefining its diameter. In that way, the NC size and density can be independently controlled by the SiO_*x*_ layer thickness and the stoichiometry parameter *x*, respectively. Several investigations seem to indicate that the ML structures do deliver on this promise. Photoluminescence (PL) spectroscopy of ML structures revealed formation of high crystalline quality Si NCs^21^,^22^,^23^,^24^, with a reduced size dispersion and superior optical properties^22^,^25^. Dedicated investigations confirmed that Si aggregation in ML structures proceeded predominantly “two-dimensionally”, within individual SiO_*x*_ layers^20^,^26^. In addition, some reports claimed that the NC size could indeed be predetermined by the thickness of the initial SiO_*x*_ layer^27^, suggesting that an independent control of NC size and density was possible. As a result, it is generally accepted that the confinement of the NCs to within the active SiO_*x*_ layers is possible. Nevertheless, these results have been contested by detailed structural characterizations of Si NCs^19^,^28^, indicating that the NC size confinement to within the thickness of the SiO_*x*_ layer, was not strictly obeyed. In light of these findings, the independent control of the NC size and concentration remains elusive. In this investigation we address these inconsistent findings. We cross-correlate structural and optical characteristics of purposefully prepared ML structures with different production parameters (SiO_*x*_ layer thickness, SiO_2_ layer thickness, SiO_*x*_ composition and annealing temperature). This multifaceted approach distinguishes the current study from the previous ones: The understanding of the combined effect of all these production parameters simultaneously, is necessary for a thorough description of NC growth in ML structures.

## Structural and optical characterization of Si NCs: limited NC growth?

For the purpose of this study, several series of Si NC samples with different characteristics are prepared. These include thick layers (500 nm) of homogeneously dispersed Si NCs as well as ML structures with various SiO_*x*_, spacer layer thicknesses and annealing temperatures. Details of the preparation procedures may be found in Methods.

We start by addressing transmission electron microscopy (TEM) and high-resolution TEM images (HRTEM), which were taken for several Si NC samples and shown in the Supplementary Information (SI). Among them, two samples, are used to investigate the structural differences between the ML structures and thick layers^29^. By TEM, we confirm the formation of equally-spaced 2-dimensional (2D) sheets of single Si clusters, with no Si precipitation appearing in the SiO_2_ spacer layers. Whereas, for the single SiO_*x*_ layer, a random dispersion of Si precipitates is observed. By HRTEM, the crystallinity and the size of the Si nanoparticles can be determined. We conclude that Si NCs of diamond structure have been created upon annealing. In order to investigate the structural differences between the ML and thick layer samples, the HRTEM images were also used to determine the NC size distribution. To obtain sufficient statistics, images of more than 100 NCs per sample have been analyzed; the resulting histograms were shown in our previous paper^29^, and are reproduced in the SI (Fig. S.1).

The following three observations can be made:

1. The mean NC size is larger in the homogeneous layer than in the ML structure^21^.
2. NC size distribution in the ML sample is narrower and more symmetric^22^, with the typical tail on the larger diameter side.
3. In the ML structure, NCs with diameters both smaller as well as larger than the initial thickness of the SiO_*x*_ layer are formed^28^.

The first two observations were previously seen as a direct result of NC confinement^21^; however the third observation – the absence of this effect – undermines such an interpretation. Actually, the combination of all three observations can be understood from the perspective of Si diffusion and precipitation. Si precipitation within the ML structures proceeds predominantly within the SiO_*x*_ layer. The lower growth rates (observation 1) can be ascribed to the lower amount of excess Si present in the ML structures, in combination with a restriction of the Ostwald ripening process^30^. The latter is then also responsible for the symmetric size-distribution (observation 2). Within this framework, the spacer layers function as buffers of pure SiO_2_, that do not contribute any Si for cluster aggregation. Locally, the amount of available Si for aggregation is decreased. The eventual NC size is defined by the production parameters (excess Si, annealing temperature) in that region, and does not follow the active layer thickness strictly (observation 3). Hence, a reduction of the NC size dispersion within ML structures does not automatically imply that the NC growth is confined to within this layer.

**Figure 1:**
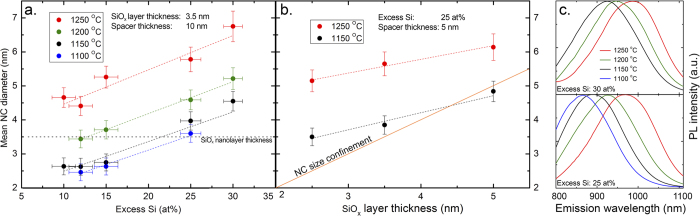
NC size characterization. (**a**) Mean NC diameter as a function of the amount of excess Si, for different temperatures estimated from PL measurements. In this plot we depict the full range of NC sizes achieved in this ML investigation for both production parameters. The mean NC size is established using the PL spectra. The linear-dependence of the NC size on the amount of excess Si is illustrated by the dotted fitting-lines. (**b**) NC diameter as a function of the SiO_*x*_ layer thickness for two annealing temperatures, also fitted with a linear dependence (black dotted lines). The orange line refers to the NC confinement model which would induce NC sizes similar to the SiO_*x*_ thickness. (**c**) PL spectra of the Si NCs samples appearing in a., with 30 at% and 25 at% of excess Si (top and bottom, respectively), for different annealing temperatures.

While we have concluded here that our high-temperature annealed (1250 °C) NCs do not exhibit size confinement, it has been suggested that such confinement is maintained while using lower annealing temperatures^28^. We investigate this by monitoring the NC growth for a fixed ML geometry upon changing the stoichiometry and the annealing conditions – shown in Fig. 1(a). Additionally, other geometries have been investigated, and the results are depicted in the SI (Fig. S.3). The mean sizes of NCs have been established from their PL spectra (shown in Fig. 1(c)), following the empirical dependence 
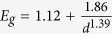
, with *E*_*g*_ and *d* being the NCs band-gap (in eV) and the NC diameter (nm), respectively, established for Si NCs prepared in the same way as done here^31^. By varying the Si excess and the annealing temperature, we were able to create ensembles of NCs with a mean size ranging from 2.4 to 6.9 nm - *i.e.* being both smaller and larger than the SiO_*x*_ layer thickness, even for the lowest annealing temperatures. We find a linear increase of the NC diameter with the Si excess, for all annealing temperatures. That a particular mean NC size can be obtained for different combinations of Si excess and annealing temperature, illustrates the overlap of the size-tuning range of these two technological variables.

In addition, we prepared ML configurations with different SiO_*x*_ layer thicknesses for annealing temperatures of 1150 °C and 1250 °C (keeping a constant spacer layer thickness of 5 nm) – shown in Fig. 1(b). These structures allow us to monitor the potential NC size confinement for different active layer thicknesses. We confirm that the mean NC size changes upon variation of the SiO_*x*_ layer thickness, in line with previous reports^17^,^21^,^23^. Nevertheless, also in the lower-temperature regime, NC size confinement, to within the SiO_*x*_ layer thickness, is not observed. We note that only once the average NC size seemingly matches the SiO_*x*_ layer thickness, (SiO_*x*_ layer thickness of 5 nm annealed at 1150 °C) which seems purely coincidental.

## Si diffusion

**Figure 2:**
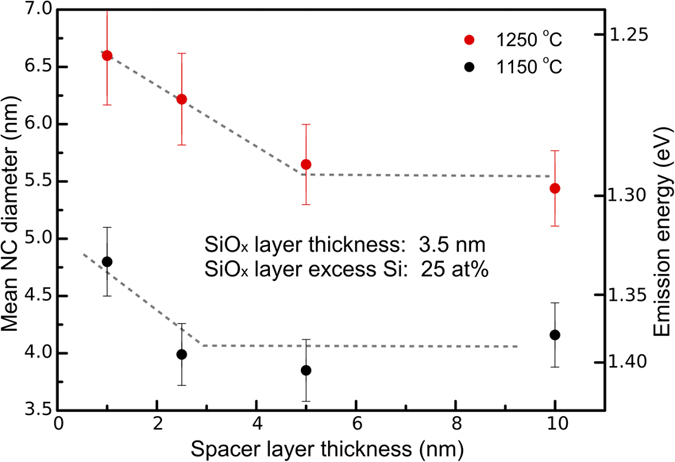
Si diffusion length. The mean NC diameter as a function of the spacer layer thickness, for annealing temperatures of 1250 °C and 1150 °C, with the dashed lines acting as guides to the eye. We interpret the changing NC diameter, as an across-spacer diffusion of silicon during NC formation. At a certain threshold thickness, the across-spacer diffusion appears to be diminished.

Our results indicate that from solely the diffusion and precipitation of Si we can explain our results, and also other observations^17^,^21^,^23^,^27^,^28^, without the need for a NC-size confinement model. Therefore, it is important to explore the role of Si diffusion inside and across the SiO_2_ spacer layers. Direct insights are obtained by investigating the formation of Si NCs in ML structures with different thicknesses of the SiO_2_ spacer layers, where the effect of Si diffusion across the spacer can be followed. We investigated PL characteristics of a series of samples for which the stoichiometry was fixed and the spacer layer thickness varied. In Fig. 2 we show the PL peak position as a function of the spacer layer thickness at annealing temperatures of 1150 °C and 1250 °C. The PL clearly shifts to shorter wavelengths as the spacer thickness grows, and then stabilizes for a sufficiently large separation between the Si NC layers. Additionally, the “final” NC diameter, as well as the threshold spacer thickness, marking the stabilization of the NC diameter, are larger for 1250 °C than for 1150 °C annealing.

While Valenta *et al.*^25^ point out the possibility of energy transfer across the spacer layer, we tend to ascribe this blue-shift to an alteration of the NC formation process, in terms of Si diffusion and precipitation, being influenced by the Si atoms which diffuse through the SiO_2_ spacer. An increase in the spacer layer thickness decreases the chance for a Si atom to cross over to the neighboring SiO_*x*_ layer. As a result, the average NC size decreases as the spacer thickness grows (resulting in blue-shifting PL spectra) with thicker spacer layers. The proposed mechanism fully accounts for the experimental findings of this study, and specifically for the annealing-temperature dependent threshold spacer thickness. Nevertheless, although the dipole-dipole interaction over the distance of the spacer layer thickness is found to be weak^29^, it cannot be excluded that also energy exchange between NCs in neighboring layers takes place.

Hence, we propose that the possibility of Si diffusion into and across the SiO_2_ spacer layers is then responsible for the lack of size confinement: NC growth within a ML structure proceeds by clustering of excess Si atoms from all directions, also from the spacer layers. The diffusion coefficient of Si within the spacer layer defines the threshold thickness of the spacers (as in Fig. 2) sufficient to prevent the “Si exchange” between neighboring SiO_*x*_ layers. In line with this reasoning, a larger threshold thickness is observed for the higher annealing temperature, a result which quantitatively coincides with the findings of Roussel *et al.*^32^, shown in the SI.

## Application potential of ML structures

**Figure 3:**
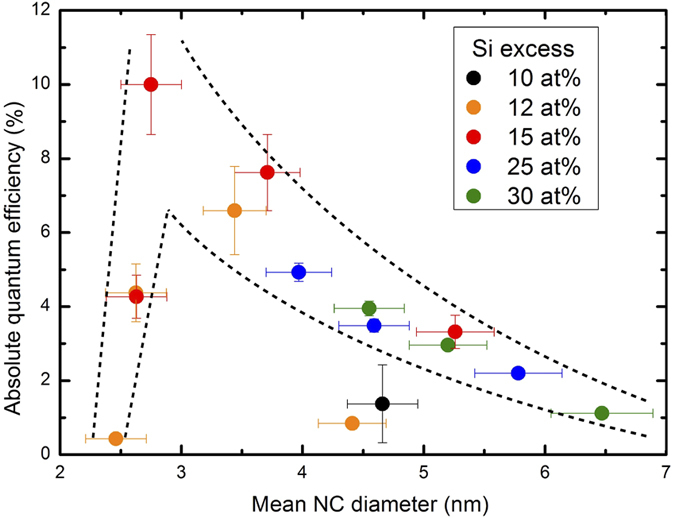
Ensemble emission efficiencies. EQE as a function of the mean NC diameter, for an excitation wavelength of 400 nm. At particular excess silicon values, different annealing temperatures have been used (ranging from 1100 °C to 1250 °C). Mean NC sizes were established using the PL spectra. Increasing efficiency values are observed for decreasing NC sizes until a threshold value of a mean NC diameter of around 3 nm. Below this size, formation of crystalline nanostructures of Si seems to be problematic, hence the emission efficiency drops.

The improved control of NC size as discussed here is pivotal for the realization of several high-potential future applications^4^, such as spectrally well-tuned LEDs and absorption spectra tuned solar cells^29^,^33^. Understanding the effect of improved NC size control is therefore vital. In Fig. 3 we show the external PL quantum efficiency (EQE), for the ML structures shown in Fig. 1. The EQE is defined here as the ratio of the number of emitted and absorbed photons^34^,^35^. This can be taken as the “figure of merit” for the quality of the NCs. The EQE values strongly peak at a particular NC size, with a gradual decrease towards bigger sizes, and a dramatic collapse for smaller sizes – similar to what has been observed for Si NCs in solution^9^ and doped Si NC systems^36^. This behavior can be explained by (i) an increased surface area, and therefore a higher probability of surface defect occurrence, for larger NCs^37^, and (ii) an increased surface curvature for the smallest NCs which results in a higher defect density^38^. The narrow size distribution of Si NC ensembles produced in ML structures, allows then to select the size range of the highest EQE, limiting contributions from both smaller and larger NCs with lower efficiency. In that way, ensembles with high EQE values could be achieved, albeit always around a similar median size.

As a side remark, the investigation of NC confinement through PL measurements fails for small NCs, with diameters smaller than ~3 nm, due to an abrupt drop of the PL EQE^9^,^36^. This has been frequently ignored in the past, and could in part explain reports on the apparent restriction of the NC size^27^.

## Conclusions

Based on structural and optical characterization of ML structures of Si NCs, prepared with different growth parameters, we have shown that the previously reported size confinement induced by the thickness of the sub-stoichiometric layer is not valid. Previous observations of such NC confinement can be explained by lower PL quantum efficiency for small NCs (below 3 nm) and should be treated as a simplified conclusion based on the observation of a smaller mean NC size and narrower size distribution. We demonstrate that the superior optical properties of ML structures can be fully accounted for by a combination of Si precipitation and diffusion, and in particular appear due to the specific “asymmetry” of the Si precipitation process which proceeds preferentially within the sub-stoichiometric layer rather than across the SiO_2_ spacers. In that respect, the spacer layer thickness, preventing the excess Si migration between the SiO_*x*_ layers, and not that of the SiO_*x*_ layer itself, becomes the most important “control” parameter of the ML structure. It is clear that the narrower size distribution of Si NCs prepared in ML structures in combination with the size-dependent EQE values, is where the added value of ML structures lies.

## Methods

For the production of samples of solid state Si NCs, excess Si was dispersed in an SiO_2_ matrix by magnetron rf co-sputtering using high purity Si (99.99%) and SiO_2_ (99.99%) targets. The sputtered films were annealed for 30 minutes at different temperatures (ranging from 1100 °C to 1250 °C) within a N_2_ environment, which induced both phase separation of Si and SiO_2_ and crystallization of the Si clusters. Information on the stoichiometry is retrieved from the calibrated sputter rates. Cross-sectional TEM and the HRTEM (JEOL JEM-200CX) images have been conducted, in the group of Fujii at the University of Kobe, further details can be found in^5^. PL spectra have been measured by a Fluorolog 2 (HORIBA Jobin Yvon) Spectrofluorometer (coupled with a Xenon lamp), incorporating both a photo-multiplier tube (PMT) and an InGaAs photo-diode for the visible and infra-red part of the spectrum, respectively. EQE measurements were performed in an integrating sphere in order to avoid possible influences of light scattering and reflections and also of directionality of emission. Xenon lamp excitation, coupled to a M130 (Solar LS) monochromator, was used to make sure that the excitation is performed in the linear absorption regime, at an excitation wavelength of 400 nm to exclude potential carrier multiplication and other size-dependent processes which could influence the EQE. All spectra were corrected for the system response.

## Additional Information

**How to cite this article**: Limpens, R. *et al.* Size confinement of Si nanocrystals in multinanolayer structures. *Sci. Rep.* **5**, 17289; doi: 10.1038/srep17289 (2015).

## Supplementary Material

Supplementary Information
