# Supplementary material for: Size confinement of Si nanocrystals in multinanolayer structures
Source: Sci Rep. 2015 Nov 25;5:17289. doi: 10.1038/srep17289 (PMC4658553; doi:10.1038/srep17289)
Supplement: Supplementary Information [file srep17289-s1.pdf]

Supplementary Information

To manuscript titled:

Size confinement of Si nanocrystals in multinanolayer structures.

By Rens Limpens, Arnon Lesage, Minoru Fujii and Tom Gregorkiewicz.

Structural characterization

In Fig. S.1 we depict the TEM and HRTEM images of 4 different samples. (a) and (b) depict the ML and single-layer samples as presented in [29], and discussed in the main text. These images were used to explore the nature of NC growth within ML structures. (c) and (d) present as-deposited and 1250 °C annealed samples which are part of the series of samples presented in Fig. S.3. We present here the TEM images of the ML structures with a thickness of 3 nm and 10 nm for the SiO_x and SiO_2 layers, respectively. These images confirm that the deposition rates are as calibrated, and that the layer thicknesses are as expected. In Fig. S.2 the histograms of the size distribution are reproduced from [29], for the samples shown in Fig. S.1(a) and (b).

NC size control in ML stacking

As we state in the main text, NC growth is not limited to the thickness of the SiO_x layer. A linear behavior of the NC diameter on the amount of Si (for all annealing temperatures) is observed for several ML configurations, even when the NC sizes approach the SiO_x layer thickness. In Fig. S.3, we depict this observation for ML structures with SiO_x layer thicknesses of 3 nm and also for thick samples ($>$ 300 nm), clearly indicating that the NC growth within ML structures is driven by the same mechanism.

Threshold spacer layer thickness

While the experimentally established threshold thickness of the spacer (Fig. 2, in the main paper) has a considerable error margin, it does complement the values obtained previously by Roussel *et al.*, [20], who estimated the necessary barrier thickness for similar samples annealed at lower temperatures (Fig. S.4). This result illustrates the essential feature of ML structures which "arrest" the volumetric 3-dimensional Si aggregation, favoring 2-dimensional diffusion within the sub-stoichiometric layer; in this aspect the spacer layer thickness becomes the essential parameter influencing size and size dispersion of the NC ensemble.

NC size determination by PL: Error calculation

The error in the determination of the NC size follows from a combination of two independently induced errors. As explained in the main text, we deduce the average NC size from the peak of the PL spectra, according to Takeoka *et al.* [31], since our samples were made in similar conditions. Nevertheless, both the estimation of the PL peak and the correlation of TEM to PL evidently suffer from uncertainties and contribute to the error of the NC size determination. The first error (position of the PL peak), is estimated as 0.01 eV by analysis of several measurements of a specific sample. In that way, we take care of experimental errors such as the resolution of the PL setup and

errors induced by changes in reflections, spot position, etc. The latter error (correlation between the PL peak and the NC size estimation from HRTEM images) is approximated by analyzing the deviation in the data presented by Takeoka *et al.* [31], and found to be ± 0.25 nm, through the whole size-regime. Subsequently, the total error is calculated following the variance formula, describing the addition of errors of independent variables. In Fig. S.5 we depict the magnitude of the error as a function of the NC size.

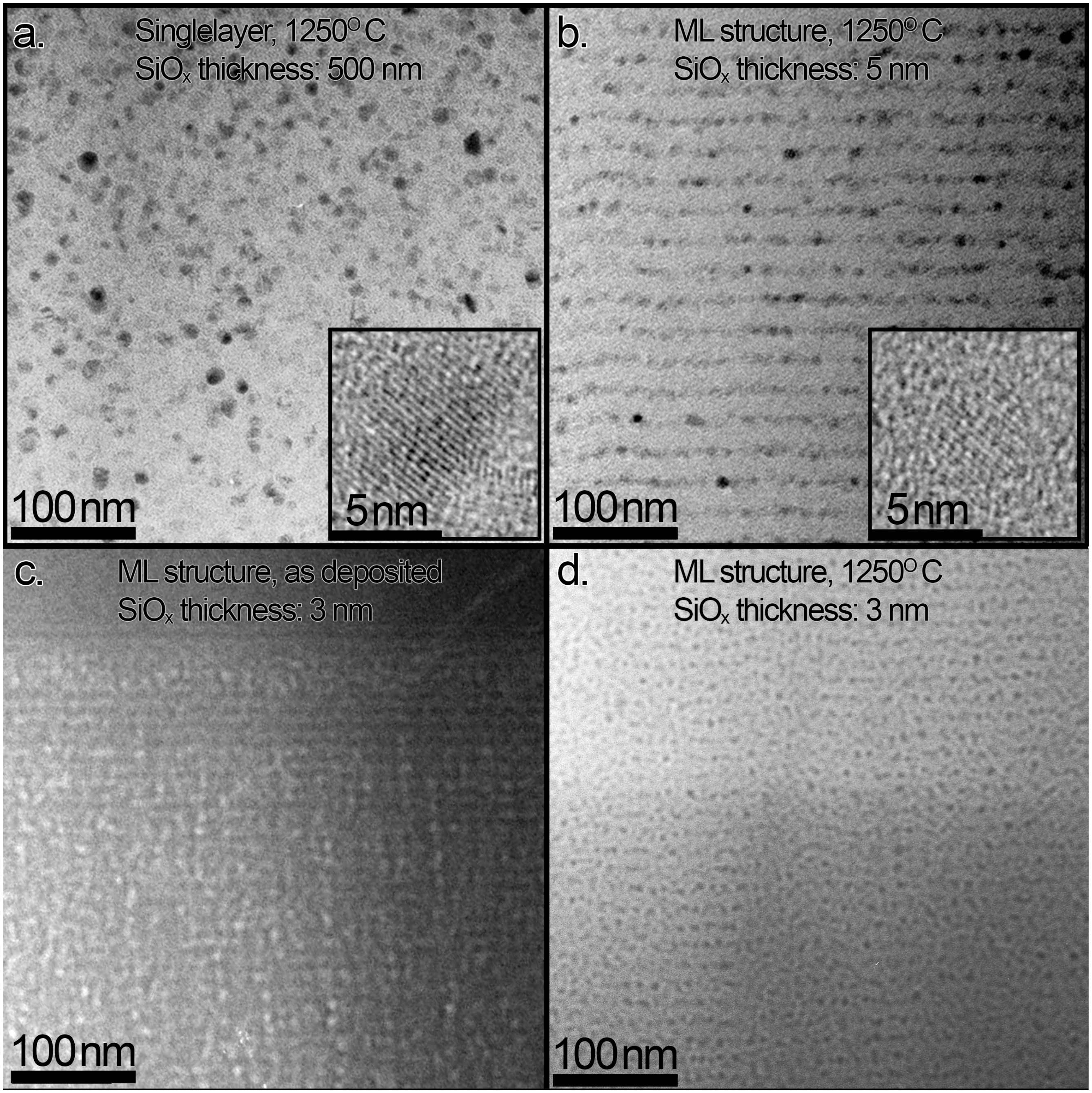

Figure S.1: **Structural characterization.** a) and b) TEM images of respectively, a single thick SiO_x layer and a multilayer samples (with identical composition and treatment) in cross-section view. The inset showing a typical HRTEM image of a NC in each of the samples. c) and d) TEM images of as deposited and 1250 °C annealed samples, with 3 nm thick SiO_x layers, respectively. The same as shown in Fig. S.3

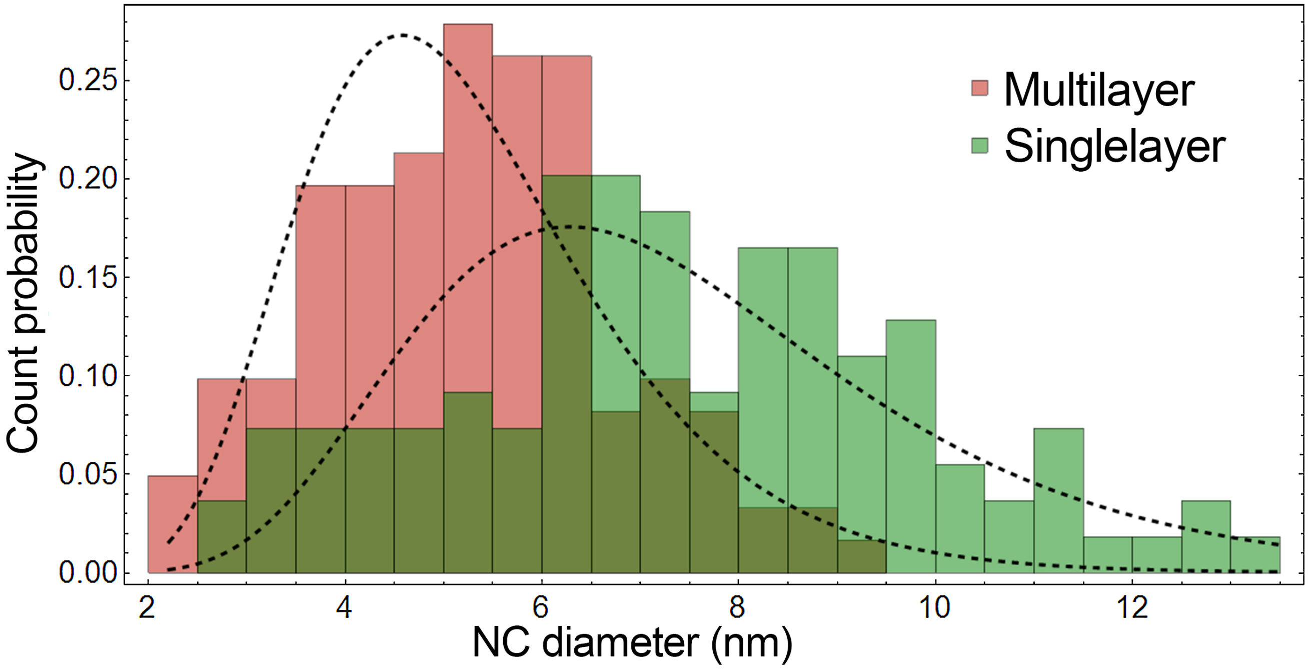

Figure S.2: **NC size histogram.** NC size histogram of the two samples shown in S.1(a) and (b), taken from the HRTEM images, with over a 100 NCs per sample. The dotted lines are log-normal distribution fits. Clearly the ML structure produces smaller sizes, with a narrower size distribution.

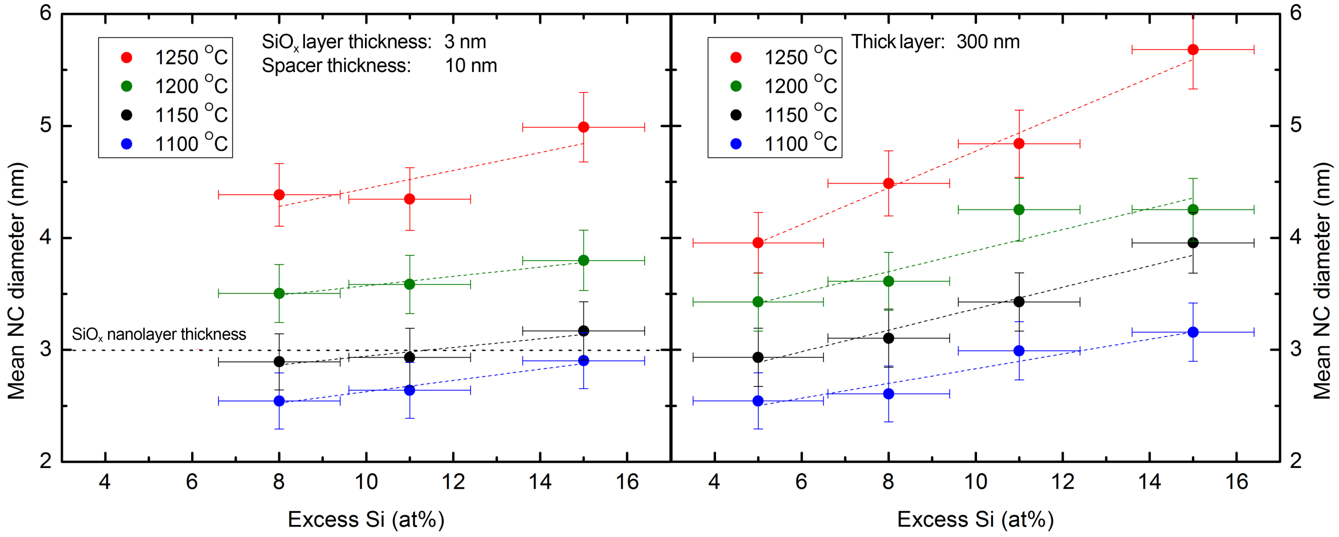

Figure S.3: **NC size characterization of several geometries.** Mean NC diameter, determined from PL characteristics, as a function of the amount of excess Si, for different temperatures. In these plots we depict the NC sizes for different structures: left, 3 nm SiO_x layer, and right, thick SiO_x layer. Both of which show similar behavior as for the 3.5 nm SiO_x layer sample in Fig. 1

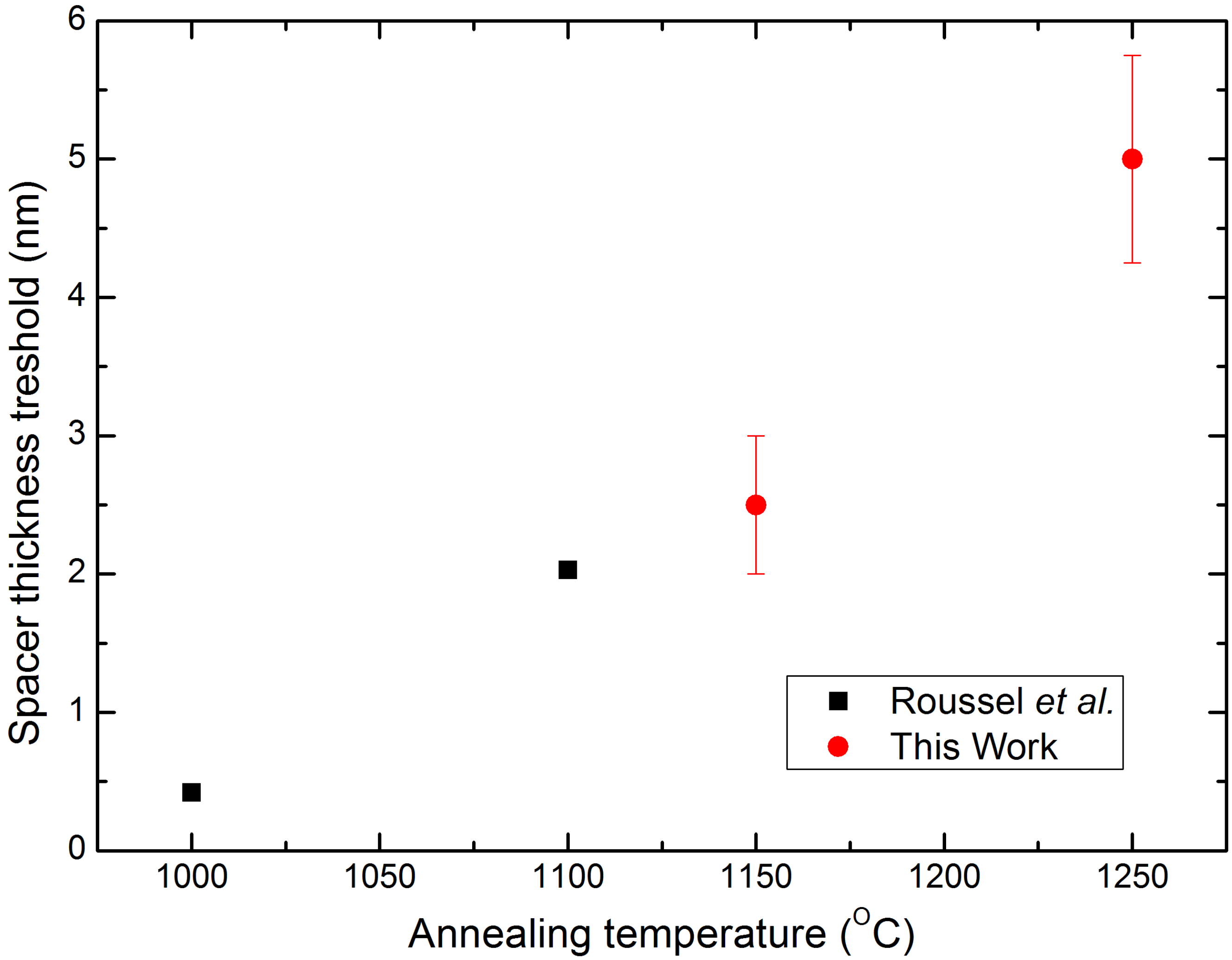

Figure S.4: **Threshold spacer layer thickness.** Our estimated threshold values for the thickness of the spacer layer are plotted together with the threshold values obtained by [20].

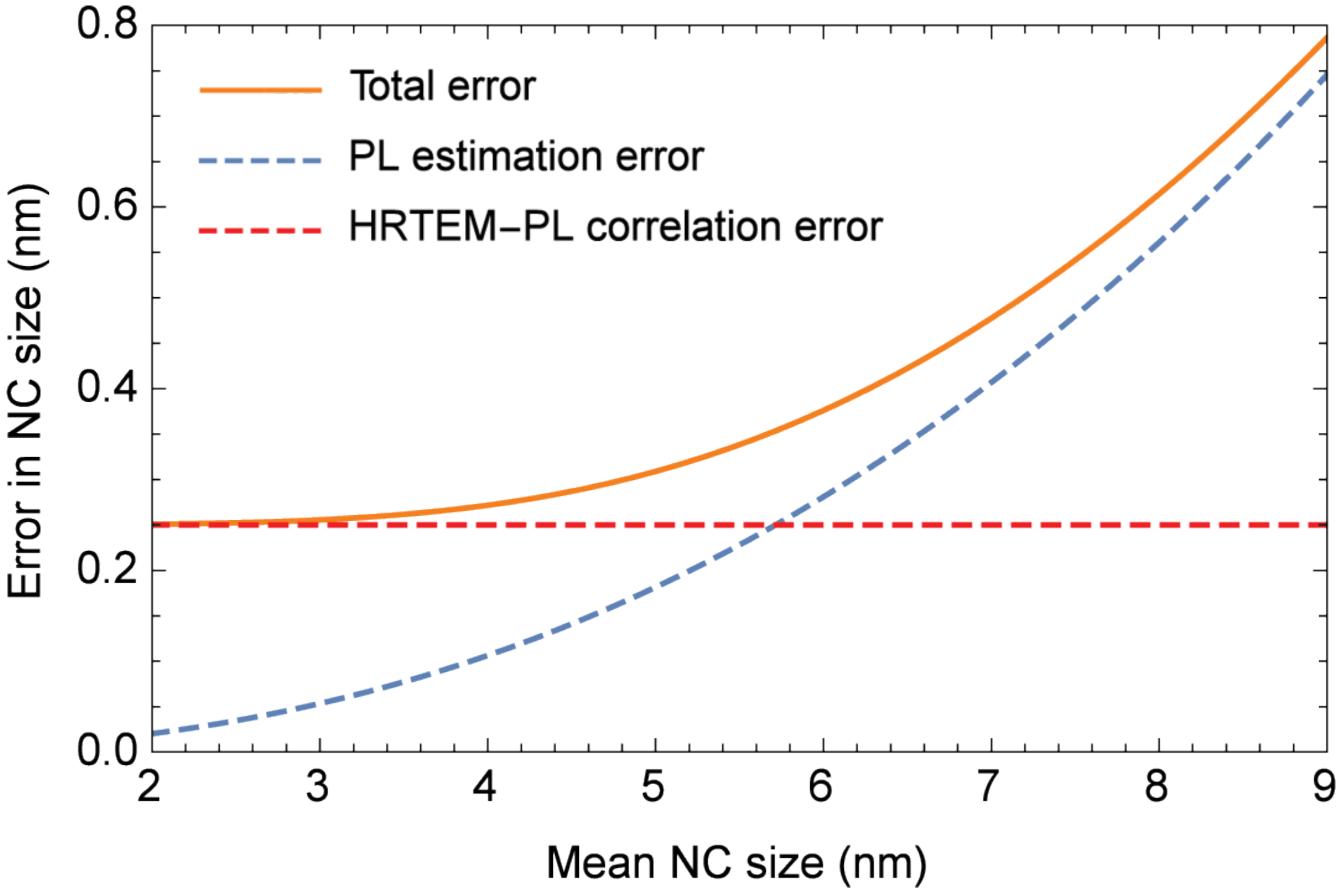

Figure S.5: **Error in the NC size.** The total error in the estimated NC size (depicted by the orange line) is made up of two components. 1) the error in the estimation of the peak of the PL spectra (given by the blue dashed curve) and 2) the uncertainty in the correlation between the actual NC size and the PL (HRTEM to PL), given by the dashed red curve.
